# Origins and characterization of variants shared between databases of somatic and germline human mutations

**DOI:** 10.1186/s12859-020-3508-8

**Published:** 2020-06-04

**Authors:** William Meyerson, John Leisman, Fabio C. P. Navarro, Mark Gerstein

**Affiliations:** 1 Computational Biology & Bioinformatics, Yale University, New Haven, CT 06511 USA; 2 Yale School of Medicine, Yale University, New Haven, CT 06510 USA; 3 Molecular, Cellular and Developmental Biology, Yale University, New Haven, CT 06510 USA; 4 Molecular Biophysics & Biochemistry, Yale University, New Haven, CT 06511 USA; 5 Department of Computer Science, Yale University, New Haven, CT 06511 USA

**Keywords:** SNVs, Somatic genomics, Germline genomics, Genomic privacy, Categorical data analysis

## Abstract

**Background:**

Mutations arise in the human genome in two major settings: the germline and the soma. These settings involve different inheritance patterns, time scales, chromatin structures, and environmental exposures, all of which impact the resulting distribution of substitutions. Nonetheless, many of the same single nucleotide variants (SNVs) are shared between germline and somatic mutation databases, such as between the gnomAD database of 120,000 germline exomes and the TCGA database of 10,000 somatic exomes. Here, we sought to explain this overlap.

**Results:**

After strict filtering to exclude common germline polymorphisms and sites with poor coverage or mappability, we found 336,987 variants shared between the somatic and germline databases. A uniform statistical model explains 34% of these shared variants; a model that incorporates the varying mutation rates of the basic mutation types explains another 50% of shared variants; and a model that includes extended nucleotide contexts (e.g. surrounding 3 bases on either side) explains an additional 4% of shared variants. Analysis of read depth finds mixed evidence that up to 4% of the shared variants may represent germline variants leaked into somatic call sets. 9% of the shared variants are not explained by any model. Sequencing errors and convergent evolution did not account for these. We surveyed other factors as well: Cancers driven by endogenous mutational processes share a greater fraction of variants with the germline, and recently derived germline variants were more likely to be somatically shared than were ancient germline ones.

**Conclusions:**

Overall, we find that shared variants largely represent bona fide biological occurrences of the same variant in the germline and somatic setting and arise primarily because DNA has some of the same basic chemical vulnerabilities in either setting. Moreover, we find mixed evidence that somatic call-sets leak appreciable numbers of germline variants, which is relevant to genomic privacy regulations. In future studies, the similar chemical vulnerability of DNA between the somatic and germline settings might be used to help identify disease-related genes by guiding the development of background-mutation models that are informed by both somatic and germline patterns of variation.

## Background

Human mutations arise in two major settings: the germline and soma. Germline mutations occur in sperm, eggs, and their progenitor cells and are therefore heritable. Somatic mutations occur in other cell types and cannot be inherited by offspring. Somatic and germline mutations matter in health and disease. Critical somatic mutations cause cancer. Somatic mutations have also been known to contribute to autoimmunity [1] and, rarely, seizure disorders [2]. Certain key germline variants cause heritable disease; and many germline inherited variants with individually small effects can have a combined [3] impact that becomes meaningful, and which may account for 30–70% [4] of the risk for common diseases.

The genomics community has produced vast, high-quality, publicly accessible databases of human variants for both the germline setting and the somatic setting. As prime examples of each: the genome Aggregation Database (gnomAD) [5] list variants from 120,000 germline whole exomes and The Cancer Genome Atlas (TCGA) [6] lists variants from 10,000 somatic whole exomes from cancer patients. While for simplicity we will refer to TCGA variants as somatic variants, it is important to emphasize that these TCGA variants arise from cancerous somatic tissues, which have different mutational processes than healthy somatic tissue, such that our results may not generalize to healthy somatic tissues.

One well-known fact about these databases is that they share many variants in common; that is, a variant defined by its chromosome, position, reference allele, and alternate allele is frequently separately listed in both gnomAD and TCGA. The latest variant re-calling effort of TCGA somatic data even includes a separate column for the germline allele frequency of somatic variants [7]. As to how these variants came to be shared between gnomAD and TCGA, however, no thorough account has been reported in the literature. Are these shared variants an expected consequence of statistically independent germline and somatic mutational processes? Or do the shared variants represent mutation hotspots common to the germline and cancerous somatic settings? Examples of this second category include the fact that spontaneous deaminations and other transitions occur at greater rates than do transversions in both the germline and soma [8, 9]. Alternatively, might some shared variants arise from sequencing errors, mislabeling of variants, or even convergent evolution?

The primary aim of this study is to investigate the origins of variants shared between gnomAD and TCGA. At a minimum, understanding the origins of shared variants will help the genomics community to better interpret these variants and use them correctly in analyses. We also characterize shared variants in terms of their distribution across subsets of germline and somatic variants to understand them in greater detail.

One lens through which we explore shared variants is in context of the similarities and differences in somatic and germline mutational processes. Mutation is a chemical reaction, and germline and somatic tissues contain DNA sequences that are virtually chemically identical. Nonetheless, germline and somatic tissues differ from each other in various ways that may affect which mutations are most likely in each setting. For example, they differ in their exposure to mutagens –with somatic skin cells being more exposed to the mutagenizing effect of UV radiation than is germline tissue [10]. Distinct somatic tissues differ from each other and from the germline in the epigenetic structure, regulation, and transcription of their genomes, which affects mutation patterns [11]. The time course over which somatic and germline variants have endured is another point of departure between these two settings: while all somatic variants from living human subjects arose within the lifetime of the sample donor, germline variants can be any age from one human generation (de novo alleles) to ancient inherited alleles. Our analysis explores shared variants in the context of these features that unite and separate somatic and germline mutational processes.

Moreover, in the course identifying the origins of shared variants, our analysis will necessarily touch on a number of questions of more general relevance in genomics. One such topic is genomic privacy. Suppose a cancer patient donates a germline sequencing sample to a research repository. If a relative of that donor then posts his or her own genetic information to an ancestry website along with his or her relationship to that donor, then from that information, any observer with access to that germline sequence of the donor can deduce the identity of the donor. In contrast, if the same donor instead donates a somatic sample consisting of de novo variants, a relative’s genetic information cannot be used to identify the donor in the same way. For this reason, it is common for somatic sequences to be open access whereas individual germline sequences are controlled access [12]. However, if enough putative somatic variants are in fact mis-called germline variants, then this would elevate the risk of re-identification of somatic samples, and could in principles affect the way the genomic community ought to process or share somatic samples. Our setup will give us a perspective on this question because in the course of identifying the probable origins of shared variants, we will attempt to estimate the number of these shared variants that arise due to true germline variants being inappropriately included in somatic call sets.

## Results

When a variant with identical chromosome, position, reference allele, and alternate allele is listed in both a somatic variant database and a germline database, we call this variant a shared variant. The goal of this work was to investigate the origins of single nucleotide variants (SNVs) shared between the largest public variant databases of human somatic exomes (TCGA) and of human germline exomes (gnomAD) and to characterize them.

### Creating a clean set of variants for analysis

To emphasize biological sources of shared SNVs, we sought to reduce the confounding influence of technical sources of shared variants. While the individual databases had been subject to strict quality control, additional artifacts become relevant in the comparison of SNVs from different whole-exome sequencing projects. Specifically, the quality of inter-project comparisons may suffer from differing exome target regions and coverage, differing variant calling and filtering rules, differing annotations of called variants, shared sequencing and mapping errors, and from samples shared between the two projects. We sought to address each of these concerns as much as practical upfront by creating a conservative whitelist of eligible exomic sites and germline and somatic variants. We restricted the whitelisted exome to the intersection of the exome target regions of each project, removed regions of known poor coverage in one project, removed sites subject to TCGA’s panel of normal filter, uniformly re-annotated all variants, removed sites known to have poor mappability, and used gnomAD’s non-cancer subset to avoid shared samples. We also removed all common germline SNVs (allele frequency > = 0.001) upfront as these sites have been subject to powerful selection forces.

An exomic site is a genomic coordinate within the whole exome sequencing target interval, which generally includes exons, untranslated 5′ or 3′ regions of mRNA, and noncoding RNA. Since at each site, one of the four types of nucleotides serves as the reference allele, there are three possible alternate alleles at each site. 24,505,884 exomic sites pass our filter, implying a set of 73,517,652 potential SNVs eligible for analysis. (Hereafter, we will use the term “*sites*” to refer to potential SNVs) Of these, 5,225,731 are observed in the germline database and 1,629,311 in the somatic database. We observe 336,987 shared variants in our clean variant list.

### Origins of shared variants

In principle, shared variants may arise from any of several reasons, including mere statistical chance, correlated mutational rates across substitution types between the germline and soma, convergent evolution, the leakage of germline variants into somatic call-sets, and shared sequencing false positives. Distinguishing the contributions of these processes is important because they have different implications for the biology of the relationship between somatic and germline exomes, the quality of sequencing, and the completeness of the genomic community’s models of human mutation.

To interpret the significance of the number of SNVs observed shared between the somatic and germline databases, it is useful to compare the number observed with the number predicted by models. The simplest model assumes that the mutation probability of a site in one data set is independent of its mutation probability in the second data set. We also consider models that assign mutation probabilities to sites based on their nucleotide context, which introduces correlations in the mutation probabilities between germline and somatic sites. When the number of observed shared SNVs differs from a model’s expectations, this excess or shortage of shared SNVs relative to the model’s predictions indicates that some additional process not included in the model is at play, and is a starting point for exploring what processes those might be.

### Shared variants are enriched nearly three-fold over expectations from statistical independence

A certain number of variants are expected to be shared between two databases by chance even if –counterfactually- the mutational processes in these databases were statistically independent from each other. The number of shared variants expected under independence, *e|independence*, is given by the equation

$$ e\mid independence=\frac{g}{n}\ast \frac{s}{n}\ast n $$

where *n* is the number of potential SNVs eligible for analysis, *g* is the number of unique eligible sites mutated in the germline database, and *s* is the number of unique eligible sites mutated in the somatic database. Using this equation, we expect 115,814 shared variants by independence. Thus, the observed number of shared variants, *b* is nearly three-fold expectations from independence, with a Forbes coefficient of association, *F* [13], of 2.910 where

$$ F=\raisebox{1ex}{$b$}\!\left/ \!\raisebox{-1ex}{$e\mid independence$}\right. $$

**Fig. 1:**
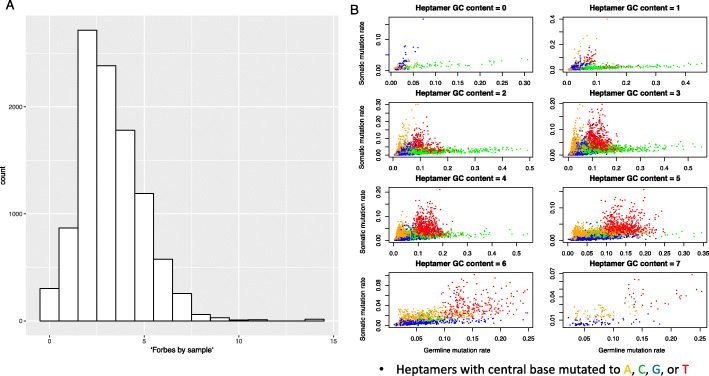
Similarities in the distribution of somatic and germline variants. **a** Histogram of Forbes coefficients across somatic samples. Virtually all somatic samples in TCGA have several-fold more variants shared with the germline database than expected by independence. **b** Mutation rates across nucleotide contexts are correlated between the somatic and germline settings. The set of all heptamers has been split by GC content and color-coded by alternate allele to show correlation structure. Heptamers involving a CpG- > T mutation are omitted from this plot for better visualization of all other heptamers

This enrichment of shared variants over expectations from independence is a pervasive phenomenon, not confined to a few outlier samples (Fig. 1a).

### A new statistical framework flexibly incorporates the distinct mutation rates of different mutation types into the expected number of shared variants

The fact that there is an excess of shared SNVs over independence is not surprising because it is known that DNA has intrinsic chemical instabilities at particular contexts. The most widely studied and prevalent chemical predispositions of DNA is that transitions occur more frequently than transversions, and that CpG sites are susceptible to spontaneous deamination to TpG [14]. These properties are true in both the germline and somatic settings. For example, in our data sets, we calculated that somatic and germline transitions, respectively, are 3.4 and 3.8 times more likely than transversions and that somatic and germline deaminations are, respectively, 19.8 and 17.2 times more likely than transversions. Therefore, our next goal was to calculate the excess of shared SNVs that cannot be explained by the greater mutation rates of deaminations and transitions over transversions. For this analysis, we introduce a new statistic, the partition-conditioned Forbes coefficient of association, *P*(*v*), which generalizes the Forbes coefficient to allow subparts of a data set to have different rates, given by

$$ P(v)=\frac{b}{\sum_{i=1}^m{e}_i} $$

where *b* is the number of shared SNVs, *m* is the number of factor levels of categorical variable *v* and

$$ {e}_i=\raisebox{1ex}{${g}_i$}\!\left/ \!\raisebox{-1ex}{${n}_i$}\right.\ast \raisebox{1ex}{${s}_i$}\!\left/ \!\raisebox{-1ex}{${n}_i$}\right.\ast {n}_i $$

and where *g*_*i*_, *s*_*i*_, and *n*_*i*_ are the number of G events, S events, and total elements of the *i*th partition of the full domain.

### Most excess shared variants can be accounted for by the greater mutation rates of deaminations and other transitions over transversions

We next applied the partition-conditioned Forbes model to identify the enrichment of shared variants relative to expectations that incorporate the distinct mutation rates of the basic substitution types. Here we set m = 3 to correspond to the three most basic types of substitution: deamination, other transition, and transversion. The calculated Forbes coefficient conditioned on a partition of sites by basic substitution type, was 1.144, indicating that 14% of variants shared between the somatic and germline databases cannot be accounted for by the database-specific relative mutation rates of deaminations, other transitions, and transversions.

### Extended nucleotide contexts exhibit mutation rates that are correlated between the germline and soma

Beyond the basic types of nucleotide substitutions (deamination, other transitions, and transversions), it has been demonstrated in the literature that more refined nucleotide contexts are also associated with distinct mutation rates. These more refined nucleotide contexts include an extended basic type [15], trinucleotide context [16], pentanucleotide context [16], and heptanucleotide context [17]. For the most part, the influence of these nucleotide contexts has been demonstrated separately in somatic and germline contexts, and what is required for these extended nucleotide contexts to influence the rate of shared variants is that the mutation rates of distinct nucleotide contexts are correlated between the germline and soma (and that these correlations are not merely driven by the more basic type of nucleotide substitutions contained within them).

As an initial step, we calculated the correlation in the mutation rate of each nucleotide context between the somatic and germline settings, finding them correlated at each level of analysis (Table 1). We find that the correlation between the somatic and germline mutation rates of different heptamers holds even when the heptamers share the same central pentamer; that is, if in the germline, TCCCCCG mutates to TCCACCG at a greater rate than ACCCCCG mutates to TCCACCG, the same will tend to hold in the soma. In Fig. 1b we graphically represent these results by plotting the mutation rate of each k-mer in the soma against the mutation rate of that k-mer in the germline.

**Table 1:** Correlation between somatic and germline mutation rates by nucleotide context

Nucleotide Context	Somatic-Germline Spearman correlation coefficient	*P*-Value
Trimer	0.71	< 2.2e-16
Trimer, excluding NCG sites	0.65	< 2.2e-16
Pentamer	0.70	< 2.2e-16
Pentamer, controlling for central 3 bases	0.33	< 2.2e-16
Heptamer	0.64	< 2.2e-16
Heptamer, controlling for central 5 bases	0.16	< 2.2e-16

### Extended nucleotide contexts explain a marginally greater proportion of excess shared variants

We hypothesized that incorporating these extended nucleotide contexts into our expectations for the number of shared variants would bring expectations closer to observations (i.e. partitional Forbes coefficients closer to 1). To test this hypothesis, we recalculated the partitional Forbes coefficient of association between somatic and germline variants by partitioning genomic sites into successively finer (longer) nucleotide contexts. We found that extended nucleotide contexts marginally explain greater fractions of the shared mutation rate (Table 2).

**Table 2:** Conditioned Forbes scores after partitioning the exome into successively finer nucleotide contexts

Model class	Partitions into which nucleotide context	Number of partitions	Number of expected shared variants	Partition-conditioned Forbes coef	Total shared SNVs explained	Incremental explanatory value
Statistical Independence	Unpartitioned	1	115,814	2.910	34.4%	34.4%
Basic mutation type	Deamination vs transition vs transversion	3	294,619	1.144	87.4%	53.0%
Extended nucleotide context	Trinucleotide	96	301,728	1.117	89.5%	2.1%
	Pentanucleotide	1536	305,511	1.103	90.7%	1.2%
	Heptanucleotide	24,576	307,393	1.096	91.2%	0.5%

### Convergent evolution does not account for the excess of shared variants

In principle, convergent evolution can result in an excess of shared variants between the soma and germline in two ways: directly and indirectly. In the direct case, convergent positive selection on the same few variants in the germline and soma would make those variants tend to be shared. In the indirect case, convergent negative selection on the same many variants in the germline and soma would make all other variants seem more shared than otherwise expected. Our analysis of the exome, in which selection forces are particularly strong, theoretically increased the risk of confounding from convergent evolution. Nonetheless, we did not expect convergent evolution to be a major explanation of the rates of shared variants we observed, because negative selection has been shown to be a much weaker force in the soma than in the germline [18], and in general the variants that are positively selected in somatic tissues are negatively selected in the germline.

To empirically test the role of convergent evolution in the observed rates of shared variants, we recalculated the shared variant rate using only synonymous variants, which are only rarely under meaningful selection pressure in humans [19]. Because of the codon code, only certain nucleotide contexts can serve as sites of synonymous variants, and these nucleotide contexts are chemically associated with distinct mutation generation rates. Therefore, for a fair comparison, we compared the shared variant rate of synonymous variants with the shared variant rate of nonsynonymous variants at sites that were matched by nucleotide context with the synonymous sites. If synonymous variants were found to have a lower shared variant rate than were nucleotide-matched nonsynonymous variants, this would be evidence in favor of convergent evolution inflating the shared variant rate at nonsynonymous sites. Instead, we observed that the synonymous shared variant rate was virtually indistinguishable from the shared variant rate of trinucleotide-matched nonsynonymous variants: Forbes coefficient of 2.649 for the synonymous subset and 2.641 for the nonsynonymous subset. These results argue against the hypothesis that convergent evolution explains a meaningful portion of the overall shared variant rate.

### Mixed evidence that a small proportion of shared variants represent germline leakage

The raw variants of a somatic sample with respect to the reference genome include both germline variants present in every cell in a given subject and somatic variants which may be unique the somatic sample obtained from that subject. In cancer sequencing projects, variants from a tumor sample are classified as somatic in origin only if they are present in the tumor sample but absent from a sample of noncancerous cells from the same subject. This digital subtraction step of somatic sequencing may not completely remove germline variants from somatic call-sets because sequencing of the normal sample is incompletely sensitive and tends to be performed at lower depth than tumor sequencing [20]. The read depth of a matched normal sample at a site is a measure of the power to exclude a leaked germline variant in that site in a somatic sample. Leaked germline variants, when present in a somatic call set, are especially likely to be shared with a germline call set. Even though we excluded TCGA subjects from our gnomAD call set, gnomAD undoubtedly contains close cousins and necessarily contains distant cousins of TCGA subjects, who inherited some of the same variants by common descent.

**Fig. 2:**
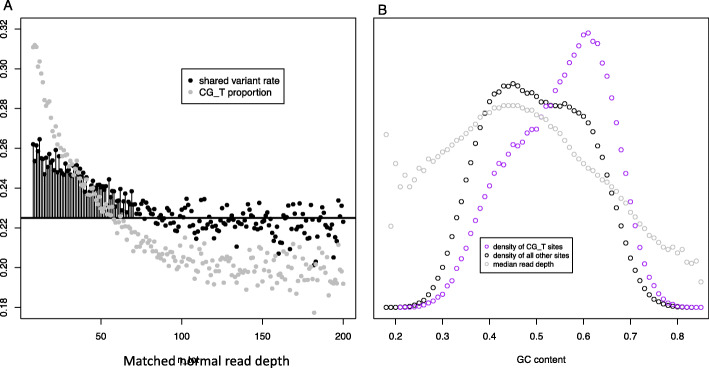
Ambiguous evidence for whether germline leakage explains some shared variants. Putative somatic variants are more likely to be shared with germline variants at sites with lower matched-normal read depth – which are just the sites with the lowest power to exclude germline leakage (**a**, black points). Alternatively, sites with lower matched-normal read-depth also happen to be sites with a high proportion of CpG dinucleotides, whose chemical instability could explain high rates of shared variants without appealing to germline leakage (**a**, gray points). In turn, the association between low read depth and high proportion of CpG dinucleotides may be related to the fact that CpG dinucleotides occur more frequently within GC-rich regions, and extreme GC content associates with low read depth (**b**)

To test whether possible leakage of germline variants in the TCGA somatic call set could explain some of the shared SNVs we observed, we performed a matched normal read depth analysis. If germline leakage is common in TCGA and leads to spurious shared variants, we would expect that the shared variant rate would be higher at sites where there was low matched normal read depth, because at such sites there is less power to exclude truly germline variants from the somatic call-set. This is indeed what we observed, with 15% more shared variants at sites with a matched-normal read depth less than 15 vs sites with a matched-normal read depth greater than 60 (Fig. 2a). If all of the enrichment of shared variants at sites of low matched normal read depth is due to leaked germline variants, we estimate that there are 2.3 leaked variants per somatic exome, higher than previous [20] estimates.

However, other lines of evidence suggest an alternative explanation of the declining shared variant rate with increasing matched normal read depth: sites of low matched normal read depth are particularly likely to be CpG sites, which have higher rates of mutation in both the soma and germline and therefore higher shared variant rates (Fig. 2a). The reason for the association of CpG sites with low read depth in turn appears to relate to the tendency of CpG sites to occur in genomic regions with high GC content, (Fig. 2b) which is a known determinant of sequencing read depth [21].

**Fig. 3:**
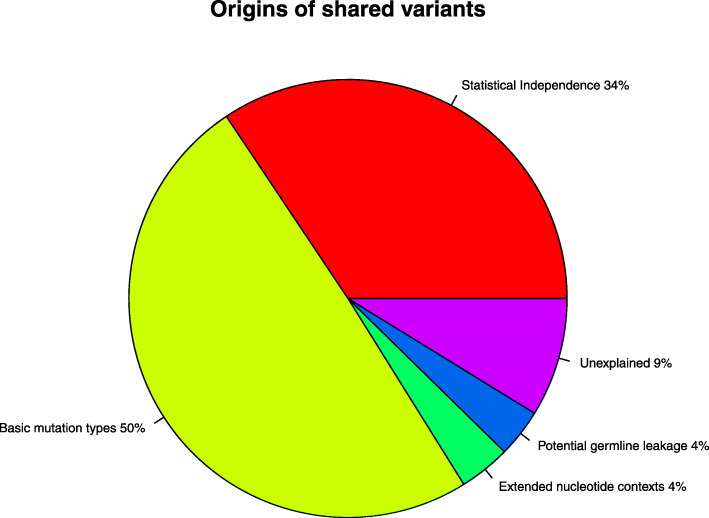
Origins of shared variants. Our best estimate of the origins of the 336,987 observed shared variants is depicted in a pie chart. Here, basic nucleotide types excludes the effects of statistical independence and the effects of the arbitrary point estimate for the number of leaked germline variants that may be correlated with basic nucleotide context

.Because of the association of GC content and thus CpG contexts with low read depth, we treat our estimate of the number of leaked germline events as an upper bound. For visualization purposes in Fig. 3, we arbitrarily assign half of this upper bound to “basic mutation types” and half to “potentially leaked germline variants.”

### False positive sequencing errors do not account for the excess of shared variants

We next tested whether sequencing errors explained the cSNV rate. Some kinds of sequencing errors consistently affect the same genomic sites, such as in repetitive regions. We had removed repetitive regions from the analysis. We also excluded sites of common germline polymorphisms, which will remove any sequencing errors that consistently affect the same genomics sites. Therefore, we focused on testing for sequencing errors that arise inconsistently. TCGA includes a validation set [22] of 24,366 somatic variants from 222 uterine corpus endometrial carcinoma samples that underwent targeted resequencing and met our filters. If shared variants result from stochastic sequencing errors, we would expect that the validation rate of shared variants would be lower than of non-shared somatic variants. Instead, we find that the validation rate of shared and non-shared re-sequenced somatic variants are indistinguishable (99.01% vs 99.03%), indicating that false-positive stochastic sequencing errors do not explain an important fraction of shared SNVs.

### Insufficient data to test association of sequencing platform with shared variant rates

We next investigated whether sequencing platform was associated with shared variant rates. All sequencing platforms have some biases, which affects the site distribution of recovered variants. We hypothesized that systematic biases in sequencing platforms leads to elevated shared variant rates when the platform of the somatic database is the same platform as for the germline database – the “*shared biases: shared variants*” hypothesis. Our ability to address this hypothesis was limited by the dominance of Illumina data in our databases.

We compared the shared variant rate of 53 legacy TCGA somatic colorectal samples sequenced using the ABI SOLiD platform against the shared variant rate with 380 somatic colorectal samples sequenced using an Illumina platform. This analysis was stratified by GC content, which is known to be related to sequencing efficiency. No adequate public cohort of ABI SOLiD germline samples was available for analysis. We observed that Illumina-sequenced somatic variants tended to be shared with Illumina-sequenced germline variants more so than did SOLiD-sequenced somatic variants tend to be shared with Illumina-sequenced germline variants, particular in genomic regions with high GC content. (Supplemental Figure 4) The “*shared biases: shared variants hypothesis*” is one potential explanation for these findings. However, because of the absence of non-Illumina platforms in our germline database and because of possible confounders, this interpretation cannot be confirmed with the available data.

### Characterization of shared variants

In the first part of our study, we showed that the main reason why somatic and germline variants are frequently shared is because certain simple types of substitutions occur at greater rates in both the germline and soma. In the second part of our study, we characterize shared variants in more detail by observing how rates of shared variants differ across biologically meaningful subsets of somatic and germline variants, and attempt to explain why the shared variant rates of these subsets differ. In this second part, we first explore subsets of somatic variants and then explore subsets of germline variants.

### Characterization of germline-shared somatic variants across distinct cancer types

#### Somatic variants from certain cancer types are especially likely to be shared with the germline database

To better characterize shared variants, we separately calculated shared variant rates by cancer type. In this analysis, we restricted the somatic call-set to a single cancer type at a time, while maintaining the full germline call-set. We observed that the rate of shared variants varies widely across cancer types from 0.11 in Lung Adenocarcinoma to 0.39 in Uveal Melanoma. (Supplemental Table 1) This observation served as a starting point for follow-up analyses aimed at explaining why somatic variants from particular cancer types are more likely to be shared with germline variants than are somatic variants from other cancer types.

#### Germline-shared variants are more common in cancer types driven by endogenous mutational processes

Different cancer types are known to have different mutational exposures, which in turn lead to nucleotide contexts having different predispositions to mutation depending on the cancer type. In the cancer genomics literature, distinct mutational exposures, such as cigarette smoke, are identified on the basis of their signatures: characteristic mutation profiles across nucleotide contexts [23]. Signatures 1 and 5 are thought to be endogenous signatures representing chemical instabilities present in any cell [24]. Because the endogenous mutational processes are believed to be active in both the germline and soma, whereas exogenous mutational processes will have distinct effects in different somatic tissues with only a very limited role in the germline, we hypothesized that cancer types with a higher proportion of variants attributed to endogenous mutational signatures would have a higher proportion of variants shared with the germline.

**Fig. 4:**
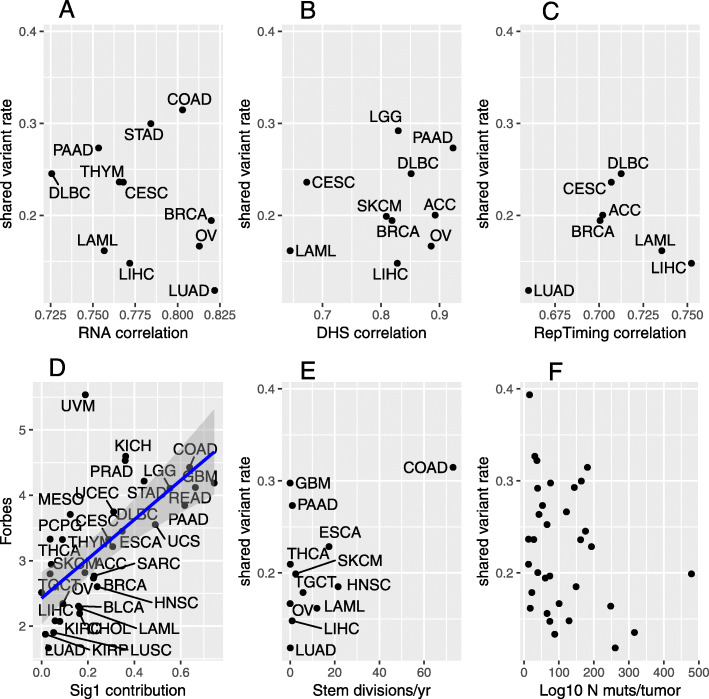
Candidate correlates of shared variant rate across somatic tissues. The similarity of three different epigenetic marks (RNA transcription, chromatin accessibility via DNase I hypersensitivity, and replication timing) between somatic tissues and a germline-like stem cell do not correlate with the rates of variants that the somatic tissues share with the germline database. **a**, **b**, **c** Somatic tissues with a higher proportion of variants attributed to the “clock-like” endogenous mutational signature 1 have higher Forbes coefficients. **d** The associations of stem cell division rate with shared variant rates and if median mutation load and shared variant rates across somatic tissues does not reach significance (**e**, **f**). For TCGA project code abbreviations, see Table 3

To test this hypothesis, we correlated the fraction of mutations in each cancer type attributable to each mutational signature with the fraction of somatic variants from that cancer that are shared with the germline [25] used non-negative matrix factorization to decompose the mutation profiles of each TCGA somatic sample into 30 recognized mutational signatures. In our analysis, using these signature contributions from [25], we found that the degree of Signature 1 is strongly related to a cancer type’s rate of shared variants (Fig. 4d). No other signature had as clear an association with the shared variant rate as did Signature 1 (Supplemental Figure 1).

**Table 3:** TCGA project codes

LAML	Acute Myeloid Leukemia
ACC	Adrenocortical carcinoma
BLCA	Bladder Urothelial Carcinoma
LGG	Brain Lower Grade Glioma
BRCA	Breast invasive carcinoma
CESC	Cervical squamous cell carcinoma and endocervical adenocarcinoma
CHOL	Cholangiocarcinoma
LCML	Chronic Myelogenous Leukemia
COAD	Colon adenocarcinoma
CNTL	Controls
ESCA	Esophageal carcinoma
FPPP	FFPE Pilot Phase II
GBM	Glioblastoma multiforme
HNSC	Head and Neck squamous cell carcinoma
KICH	Kidney Chromophobe
KIRC	Kidney renal clear cell carcinoma
KIRP	Kidney renal papillary cell carcinoma
LIHC	Liver hepatocellular carcinoma
LUAD	Lung adenocarcinoma
LUSC	Lung squamous cell carcinoma
DLBC	Lymphoid Neoplasm Diffuse Large B-cell Lymphoma
MESO	Mesothelioma
MISC	Miscellaneous
OV	Ovarian serous cystadenocarcinoma
PAAD	Pancreatic adenocarcinoma
PCPG	Pheochromocytoma and Paraganglioma
PRAD	Prostate adenocarcinoma
READ	Rectum adenocarcinoma
SARC	Sarcoma
SKCM	Skin Cutaneous Melanoma
STAD	Stomach adenocarcinoma
TGCT	Testicular Germ Cell Tumors
THYM	Thymoma
THCA	Thyroid carcinoma
UCS	Uterine Carcinosarcoma

### No evidence that epigenetic features explain the differences in rates of germline-shared variants across cancer types

Another key way in which somatic tissue types differ from each other is in their epigenetic architecture. It is well-established that certain epigenetic features –include DNA replication timing, chromatin accessibility, and RNA transcription- are associated with mutation rates [11]. Therefore, we hypothesized that somatic tissues with an epigenetic architecture similar to that of the human germline would have higher rates of variants shared with the germline.

To test this hypothesis, we correlated the distribution of DNA replication timing, DNAse hypersensitivity (as a marker for chromatic accessibility), and RNA transcription between each somatic tissue and a proxy for the germline. DNA replication timing values and DNAse hypersensitivity scores per megabase were obtained from ROADMAP Epigenome [26]. RNA transcription values per gene were obtained from GTEX [27]. As a proxy for the germline, we used the human embryonic stem cell line H1. Cancer types were matched to corresponding Roadmap or GTEX tissues according to Supplemental Table 1.

Contrary to our hypothesis, somatic tissues whose epigenetic features were better correlated with the features in the germline proxy did not have higher rates of germline-shared variants (Fig. 4a, b, c). Notably, the epigenetic features of most somatic tissues had a similar correlation coefficient with the germline proxy, which limited the sensitivity of this approach. A further limitation of our approach is that we used aggregate data from approximately-matched somatic tissues.

### No evidence that replicative capacity by cell-of-origin explains the differences in rates of germline-shared variants across cancer types

A further way in which somatic tissues differ from each other is that the stem cells of some somatic tissues divide more quickly than those of others. All things being equal, more rapidly dividing tissues might be expected to have a higher ratio of endogenous mutations from DNA replication errors to exogenous mutations. As these somatic endogenous mutations may tend to arise at similar genomic sites as germline endogenous mutations, it could be hypothesized that somatic variants from faster-dividing somatic tissues would be more likely to be shared with the germline than those from slower-dividing somatic tissues.

To test this hypothesis, we obtained estimates of the rate of division of stem cells for various tissues from [28] and matched them to TCGA cancer types (Supplemental Table 1). We found a positive trend for faster-dividing somatic tissues to have higher rates of germline-shared variants, but this trend was not statistically significant (*p* = 0.09 by Pearson significance test) (Fig. 4e). A limitation of this approach is that the cell-of-origin for some cancer types, such as Glioblastoma Multiforme is uncertain [29].

### .Characterization of somatically-shared germline variants across germline allele frequencies, allele ages, and ancestries

#### Recent germline alleles, including de novo germline variants, are more likely to be somatically-shared

One major difference between somatic and germline variants is that, while all somatic variants from living humans arose recently -within years to decades of sequencing-, some ancient germline variants have been passed on for hundreds of millennia. Not all germline variants are so ancient: de novo variants, for example -which pervade an organism’s cells but are present in only a gamete lineage in a parent- are practically as recently-derived as are somatic variants. Between these two extremes of de novo variants and ancient variants, germline variants comprise a whole spectrum of allele ages.

Alleles that have withstood the test of time might be expected to collect around a different set of genomic sites than do newer alleles because of the accumulated influence of long-term mutational forces, including biased gene conversion (in which greater hydrogen bonding between G and C leads to G and C alleles being preferentially passed on at AT/GC heterozygous sites) [30] and evolutionary selection pressure (noting that, although we observed that evolutionary selection pressure does not explain the rate of shared variants overall, it may still affect the small subset of ancient variants). Therefore, we hypothesized that recently-derived germline variants are more likely to occur at the same sites of necessarily-recent somatic variants –i.e. have a higher somatically-shared rate– than do ancient germline variants.

**Fig. 5:**
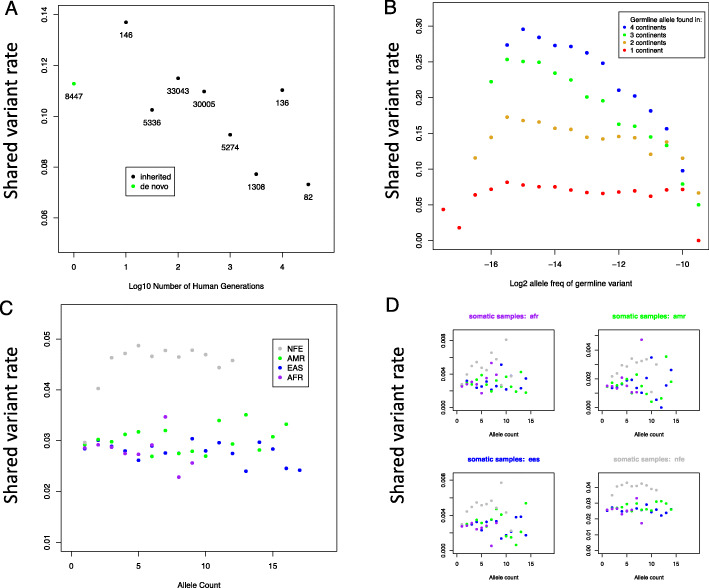
The shared variant rate of germline variants by allele age and ancestry. More recently-derived germline variants, including de novo variants, have higher rates of being somatically-shared. Points are labeled according to the number of variants within the age bin corresponding to each set of variants. **a** Ultra-rare germline variants present on multiple continents have higher rates of being somatically-shared. **b** Among germline variants unique to one continental ancestry in our data set, variants from donors of European ancestry have higher rates of being shared with the somatic database than do variants from any other continental ancestry. **c** Exclusively European germline variants have elevated rates of being shared with somatic samples of any continental ancestry, and no other germline continental ancestry has a markedly higher shared variant rate when comparing with somatic variants of each continental ancestry. (D) NFE = Non-Finnish European; AMR = Admixed American; EAS = East Asian; AFR = African or African American

To test this hypothesis, we analyzed the relationships between a germline variant’s allele age and its probability of being somatically-shared. We annotated germline variants with their allele ages taken from the Atlas of Variant Age, which uses coalescent models to estimate ages of germline variants in public variant databases [31]. For this analysis, we also incorporated 8447 variants from denovo-DB which were confirmed through parent-offspring trio sequencing to be de novo in origin, representing an allele age of <= 1 generation [32]. We find that germline alleles < 500 generations old have substantially higher somatically-shared rates than do germline alleles > 500 generations old, (*p*-value 8.7e-09 by proportion test) (Fig. 5a).

These results are consistent with a model in which long-term mutational forces over time shift the genomic distribution of germline variants from sites prone to mutation-generation in both the germline and soma, to genomic sites at which mutations endure. These results also predict that rare examples of ancient somatic variants, such as the 11,000-year-old somatic variants from the canine venereal transmissible tumor [33], might have a higher rate of being shared with older canine germline variants than with recent canine germline variants.

#### Ultra-rare germline variants present on multiple continents are especially likely to be somatically-shared

Our larger hypothesis is that the key reason that germline and somatic variants tend to fall on the same sites is that some of the same sites are mutation-prone in both the germline and somatic settings. One way we tested this larger hypothesis was through an analysis of the role of nucleotide context in shared germline and somatic variants. This approach was successful because nucleotide context is well-established as a feature associated with mutation rates. We also pursued a completely different approach, in which sites are identified as mutation-prone within the germline not in virtue of the sites’ genomic features but by evidence of mutational recurrence within that setting at the site. Put another way: one sign that a genomic site is mutation-prone in the germline is if it has arisen independently in the germline multiple times.

Merely observing that a variant is present in the germline of two or more individuals does not suffice to show that the variant has independently arisen multiple times, because such cases often reflect the inheritance from a common ancestor of a variant that arose once. Moreover, while oceans and mountains reduce the extent of shared inheritance of alleles among individuals from different continents, high-frequency alleles have many opportunities to spread from continent to continent through human migrations. However, for multi-continental germline alleles with low frequency in the human population, opportunities for trans-continental migrations are more limited, so we reasoned that these variants would be enriched in variants that have independently arisen and therefore be mutation-prone and more likely to also be present in somatic samples.

To test this hypothesis, we calculated the rate that germline variants are somatically-shared across germline allele frequencies, after stratifying by the number of continents on which the germline variants are found. Consistent with this hypothesis, we found that for ultra-rare germline variants, the somatically-shared rate rises with the number of continents on which the variants are found (Fig. 5b). The intended interpretation of this finding is that the most mutation-prone germline sites are also prone to mutation in the soma. An alternative interpretation of this observation is that ultra-rare variants present on multiple continents represent sequencing errors that are shared with errors in the somatic sequencing project.

#### Ultra-rare germline variants of European ancestry are more frequently somatically-shared

We next wished to test whether the rate that a germline variant is somatically shared varies by continental ancestry. We calculated the somatically-shared rate of germline variants across a range of ancestries and allele counts. Among ultra-rare germline variants, those of European ancestry were more frequently somatically-shared (Fig. 5c). Because most somatic samples were from European donors, we initially hypothesized that the greater degree of somatically-shared variants of European germline variants could be related to the leakage of Europe germline variants into European somatic samples. To test this hypothesis, we separately calculated the shared variant rate between each combination of germline continental ancestry and somatic continental ancestry. Under the germline contamination hypothesis, we expected to see a higher rate of shared variants when germline variants of one ancestry were compared to somatic variants from donors of the same ancestry. Contrary to this hypothesis, European germline variants had a higher rate of being shared with somatic samples from donors of any ancestry, and African, East Asian, and Admixed American variants did not have elevated rates of being shared with somatic samples from donors of those respective ancestries (Fig. 5d). At present, the higher rate of shared variants among ultra-rare germline variants from donors of European ancestry is an unexplained observation.

### Comparison of shared variants to variants unique to one database

We next sought to characterize how variants shared between the somatic and germline call-sets compare with variants unique to one call-set along a range of dimensions. In one analysis, we compared the variant allele frequency (VAF) distribution of shared somatic variants vs that of unique somatic variants. In general, high VAF variants (VAF 0.3–0.5) are variants supported by many reads in the tumor and represent variants that arose during the earlier stages of a tumor’s evolution, whereas low VAF variants are supported by fewer reads and arose later in a tumor’s history. We found that shared variants tend to have higher VAFs than do unshared somatic variants. (Supplemental Figure 2) The reason for this association is unknown. One possible technical explanation is that shared variants have a higher rate of being leaked germline variants, which will tend to have high VAFs close to 0.5, which is the expected VAF of a heterozygous germline mutation. A possible biological interpretation is that, because low VAF variants arise later in a tumor’s course, they arise in the cancer cell at a time of greater genomic instability, which leads to divergent mutation distributions compared with those of the germline setting.

We also compared shared variants with germline-unique and somatic-unique variants along the dimensions of regional GC content, regional CpG content, background selection from [39], RNA expression from [40], and germline pathogenicity (CADD) scores from [19]. Prior to performing these calculations, we down-sampled the variant lists such that each variant category (shared, germline-unique, and somatic-unique) had identical trinucleotide context distributions to reduce confounding. We found that the regional GC content and RNA expression levels of shared variants was closer to that of somatic variants than of germline variants. Alternatively, the germline pathogenicity score (CADD) of shared variants was closer to that of germline variants than of somatic variants. Meanwhile the background selection score of shared variants was appreciably larger than those of both somatic and germline variants and the regional CpG content of shared variants was appreciably lower than those of both somatic and germline variants (Supplemental Figure 3).

## Discussion

Our primary aim was to identify the origins of variants shared between the germline database gnomAD and the cancerous somatic database TCGA. We focused on single nucleotide variants because of the greater density of single nucleotide data. A simple model that assumes statistical independence between germline and somatic mutational processes can explain only one third of the observed share variants. A model that incorporates basic nucleotide context in additition can explain 87% of shared variants, and a model that incorporates extended nucleotide context can explain 91% of shared variants. Leaked germline variants present a mixed picture and could have contributed to as much as 7% of germline variants, but this requires strong assumptions. Neither stochastic sequencing false positives nor convergent evolution explain a large portion of shared variants. The remaining shared variants that cannot be accounted for by extended nucleotide context could possibly result from shared epigenetic features, but our limited explorations did not find evidence of this. Other technical factors such as broad patterns in coverage, or biological factors such as periodicity in the histone code could potentially explain this small unexplained excess, but were not tested.

Further characterizing shared variants revealed a number of patterns. Cancer types different in their rate of germline-shared variants, with cancer types driven by endogenous mutational processes having higher rates of germline-shared variants. Recently-derived germline alleles are more likely to be somatically-shared than are ancient germline alleles, which may relate to long-term forces of selection and drift acting to a greater cumulative extent on ancient germline alleles than on recent germline alleles and necessarily recent somatic alleles. Germline variants from donors of various ancestries were just as likely to be somatically-shared, with the exception of ultra-rare European variants which had higher rates of being somatically-shared for unknown reasons.

Despite major differences, there are substantial similarities in the mutational forces that influence somatic and germline variants. Future studies could potentially capitalize on these similarities by, for example, using germline SNV density as one feature for calibration expectation for somatic mutation rates by genomic segment.

Neutral models that rely only on nucleotide context capture a large but incomplete fraction of all forces affecting mutation rates. This provides some support for the notion that nucleotide context is a sufficient model feature when coarse mutation rate expectation are all that are required while additional genomic and epigenetic features should be employed when fine models of mutation rate are desired.

A possible conclusion at the outset of this study, were it to turn out that many shared variants represent true germline variants mis-called as somatic, would be that TCGA somatic call-sets may require further measures to ensure the privacy of somatic donors, either through deindividuation or further filtering. However, we did not find any compelling evidence that shared variants are often misclassified germline variants. Instead, we found mixed evidence that some shared variants might be misclassified germline variants, but that technical artifacts due to extreme GC content could similarly account for our observations.

One key limitation of this study, like many genomic studies, is that it is observational in nature and therefore not well-suited to isolating causation. In attempting to find the origins or shared variants, we are able to uncover associated features but cannot verify that these features are fundamentally causal and in some cases, the identified biological features be only spuriously associated with shared variants through correlation with technical features. Nonetheless, the basic types of nucleotide context in particular have been shown to have a causal role in mutation rates.

One choice in this study that limits the generalizability of findings is the confinement of analysis to exomic regions. This decision was made because of the greater density of SNVs in public exome databases over public. An implication of this choice is that it was not practical to test the role of genomic region on shared SNV rates. The focus on exonic regions also increases the theoretical potential of confounding influence of evolutionary selection pressure, but our results did not suggest an important role of evolutionary selection pressure in explaining shared variants.

Another choice we have mentioned previously is the use of somatic variants primarily from cancerous tissues. This choice reflects the far greater availability of cancerous over non-cancerous somatic data, which in part reflects clinical priorities and in part technical feasibility. As a result, some findings that relate germline variants to cancerous somatic variants may not apply in full to relating germline variants to non-cancerous somatic variants.

## Conclusion

The 336,987 filtered variants that are shared between gnomAD and TCGA likely mostly represent true biological variants that arose independently in the human germline and human soma. 87% of these variants came to be shared because of the greater rate at which deaminations and other transitions occur over transversions in both the germline and soma. Our evidence indicates that these variants are usually appropriate to use in ways similar to non-shared variants in downstream applications.

## Methods

All statistics were computed using R (version 3.5.1, R Development Core Team, 2018).

### Data and processing

We used the largest public databases of somatic and germline variants, and applied conservative filters to reduce the impact of technical bias. For the somatic database, we used the public whole exome somatic call-set from The Cancer Genome Atlas (TCGA) of 10,221 cancer patients [7]. For the germline database, we used the public whole exome germline call-set from the Genome Aggregation Database (gnomAD) of 125,748 human subjects [5]. The GATK VariantsToTable tool was used to extract the non_cancer subset of gnomAD, which was used in all analyses involving gnomAD [34].

Some parts of the analysis were also attempted on smaller but conceptually cleaner databases. For example, Denovo-db served as an additional germline database. Moreover [22, 32], supplied additional technical replicates of somatic samples.

Analysis was restricted to potential SNVs that are free from known technical liabilities. To accomplish this, we started with a set of all possible SNVs of the reference genome hg19 and filtered down to a conservative universe of potential SNVs. Only autosomes were included because of possible sex imbalances between data sets. To minimize artifacts due to mapping errors, we excluded sites that overlapped the EncodeDac or EncodeDuke mappability blacklists [35], that are predicted to be not uniquely alignable with 24 base pair reads. Similarly, we removed sites that fall in repetitive regions such as genomic super duplications, simple repeats, and microsatellites, or that are otherwise flagged by RepeatMasker [36].

To minimize artifacts due to non-uniform exome capture and coverage, we restricted sites to the intersection of the exome interval lists of gnomAD and TCGA, required sites to have 20 or more reads in 50% of gnomAD samples, and excluded sites in which fewer than 30% or more than 70% of the surrounding 100 bases are a G or C. To minimize artifacts related to germline contamination and sequencing error hot-spots, we removed sites with a gnomAD allele frequency of 0.1% or greater. Removing sites of common human polymorphisms also had the advantage of effectively reducing discrepancies between hg19 and the human ancestral genome.

Additionally, we only included SNVs from gnomAD graded “PASS.” For TCGA, we excluded any SNV with the filter “nonpreferredpair” or “oxog.” For denovo-db, we only included variants that were obtained through whole exome sequencing.

In the main analysis, we mark filtered SNVs as being present or absent in a database, initially ignoring allele count. When characterizing germline variants in greater detail, we did make use of ancestry-specific allele counts and allele frequencies.

### Application of partitional Forbes statistic

To apply the partitional dependence metric on genomic data, it was necessary to divide the genome into partitions defined by a shared level of a categorical variable. We repeated the analysis for various different partitions of interest, using a partitioning scheme based on nucleotide context. For example, every possible SNV can be grouped into one of 12 basic types of nucleotide context: A- > C, A- > G, …, T- > C, and T- > G, which defines a complete partition of the genome eligible for analysis through the partitional dependence framework. Extended nucleotide contexts, which includes 1 or more flanking bases, formed finer partitions of the genome and were also analyzed up to 3 flanking bases on either side. We used the Rsamtools [37] and GenomicRanges [38] R packages to extract the adjacent nucleotides for each potential variant, which were concatenated around the reference and alternate allele. When defining nucleotide context-based partitions, reverse complements were collapsed onto a central pyrimidine; for example A [G- > T]C was considered an instance of G [C- > A] T since wherever one of these trimers is present on the positive strand of the genome, the other trimer occurs on the negative strand of the genome.

### Estimating germline contamination

To estimate the number of germline contaminants among shared SNVs, we took the baseline shared variant rate to be the shared variant rate among somatic variants with at least 200 reads in the matched normal, which visually approaches the asymptotic behavior of shared variant rate with read depth. For read depth less than 98, each read depth was found to be associated with a higher shared variant rate than at a read depth of 200, which for purposes of this estimate we assumed was due to leaked germline variants. Then for each matched normal read depth less than 98, we calculated the number of variants that would be expected to be shared using the baseline shared variant rate. We subtracted the number of baseline-expected shared variants from the observed number of shared variants for these variants with low read depth to estimate the total number of extra shared variants that may result from germline contamination.

## Supplementary information

**Additional file 1: Supplemental Figure 1.** Association between the loading of each mutational signature and the shared variant rate across somatic tissues. Each panel represents a distinct mutational signature; 1–30 from [25]. Each point represents a different somatic tissue. The shared variant rate of somatic samples are plotted on the y-axis against the proportion of variants in that sample that can be attributed to the given signature. At the top of each panel is listed Pearson’s Rho for the association between the signature’s loading and the shared variant rate across samples. Signature 1 has the greatest in magnitude association with shared variant rates

**Additional file 2: Supplemental Figure 2.** The somatic variant allele frequency (VAF) distribution of somatically-unique and germline-shared somatic variants.

**Additional file 3: Supplemental Figure 3.** Comparison of shared variants with somatically-unique and germline-unique variants along a range of genomic dimensions. Depending on the genomic dimension studied, shared variants better resemble either somatic values or germline values – or behave their own way. Units for each variable are scaled to make the set of all variants that are either somatic-unique or germline-unique have mean 0 and standard deviation 1.

**Additional file 4: Supplemental Figure 4.** Somatic sequencing platform and germline-shared variant rates. The rates at which somatic variants from 52 ABI-SOLiD sequenced colorectal tumors and 380 Illumina sequenced colorectal tumors are shared with an Illumina sequenced germline database, stratified by GC content bin. The size of each point is proportional to the logarithm of the number of variants that fall within each GC content bin.

**Additional file 5: Supplemental Table 1**. Manual matching of TCGA cancer types to tissue samples from GTEX, ROADMAP, ENCODE, and Tomasetti et al.

## Data Availability

All core data analyzed during the current study are publicly available. Germline variants and coverage information from 123,136 whole exomes was obtained from the genome Aggregation Database (gnomAD) version 2.0.2 https://storage.googleapis.com/gnomad-public/release/2.0.2/vcf/exomes/gnomad.exomes.r2.0.2.sites.vcf.bgz and https://storage.googleapis.com/gnomad-public/release/2.0.2/coverage/combined_tars/gnomad.exomes.r2.0.2.coverage.all.tar. Somatic variants for 10,221 whole exomes of cancer patients was obtained from The Cancer Genome Atlas and is freely publicly available from https://api.gdc.cancer.gov/data/1c8cfe5f-e52d-41ba-94da-f15ea1337efc. We obtained the targeted deep-sequencing validation cohort of somatic samples from https://tcga-data-secure.nci.nih.gov/tcgafiles/tcgajamboree/tcgajamboree/mc3/validation_data/ucec/ucec_3_center_222_cases_vars_within_capture_targets_with_validation_status.maf.gz which are available to qualified researchers who apply for access. De novo germline variants were obtained from denovo-db at http://denovo-db.gs.washington.edu/denovo-db.non-ssc-samples.variants.vcf.gz. gnomAD target regions: https://storage.googleapis.com/gnomad-public/intervals/exome_calling_regions.v1.interval_list. TCGA (MC3) target regions: https://api.gdc.cancer.gov/data/7f0d3ab9-8bef-4e3b-928a-6090caae885b.

## Abbreviations

SNV: Single nucleotide variant
TCGA: The Cancer Genome Atlas of somatic variants from cancer patients
gnomAD: The genome Aggregation Database of germline variants from a range of healthy subjects and patients without Mendelian disease
