# Supplementary material for: Origins and characterization of variants shared between databases of somatic and germline human mutations
Source: BMC Bioinformatics. 2020 Jun 4;21:227. doi: 10.1186/s12859-020-3508-8 (PMC7273669; doi:10.1186/s12859-020-3508-8)
Supplement: Supplementary file 1 — Additional file 1: Supplemental Figure 1. Association between the loading of each mutational signature and the shared variant rate across somatic tissues. Each panel represents a distinct mutational signature; 1–30 from [25]. Each point represents a different somatic tissue. The shared variant rate of somatic samples are plotted on the y-axis against the proportion of variants in that sample that can be attributed to the given signature. At the top of each panel is listed Pearson’s Rho for the association between the signature’s loading and the shared variant rate across samples. Signature 1 has the greatest in magnitude association with shared variant rates [file 12859_2020_3508_MOESM1_ESM.pdf]

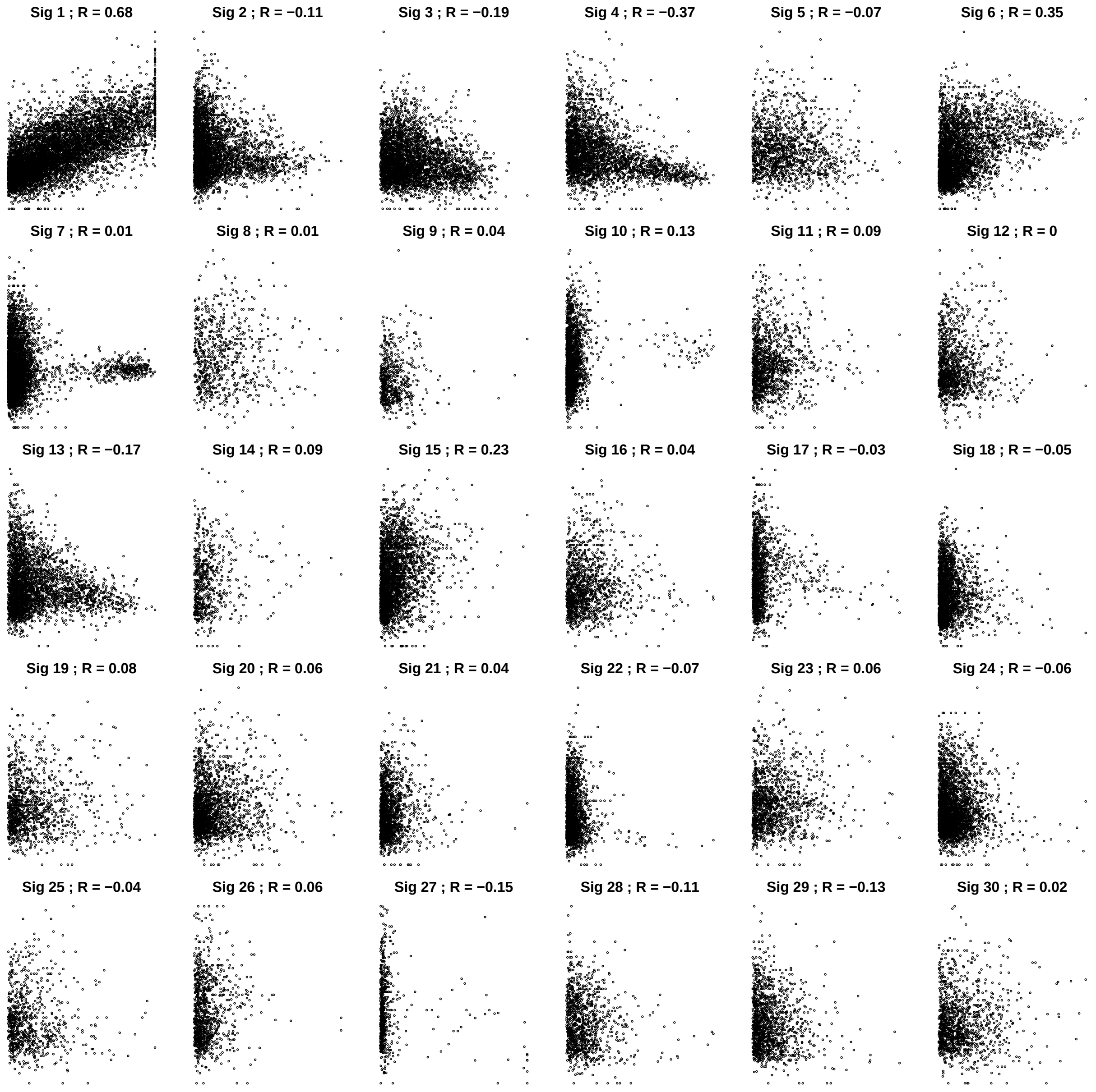
Sig 1 ; R = 0.68
Sig 2 ; R = −0.11
Sig 3 ; R = −0.19
Sig 4 ; R = −0.37
Sig 5 ; R = −0.07
Sig 6 ; R = 0.35
Sig 7 ; R = 0.01
Sig 8 ; R = 0.01
Sig 9 ; R = 0.04
Sig 10 ; R = 0.13
Sig 11 ; R = 0.09
Sig 12 ; R = 0
Sig 13 ; R = −0.17
Sig 14 ; R = 0.09
Sig 15 ; R = 0.23
Sig 16 ; R = 0.04
Sig 17 ; R = −0.03
Sig 18 ; R = −0.05
Sig 19 ; R = 0.08
Sig 20 ; R = 0.06
Sig 21 ; R = 0.04
Sig 22 ; R = −0.07
Sig 23 ; R = 0.06
Sig 24 ; R = −0.06
Sig 25 ; R = −0.04
Sig 26 ; R = 0.06
Sig 27 ; R = −0.15
Sig 28 ; R = −0.11
Sig 29 ; R = −0.13
Sig 30 ; R = 0.02
