# Supplementary figures and images for: Origins and characterization of variants shared between databases of somatic and germline human mutations
Source: BMC Bioinformatics. 2020 Jun 4;21:227. doi: 10.1186/s12859-020-3508-8 (PMC7273669; doi:10.1186/s12859-020-3508-8)

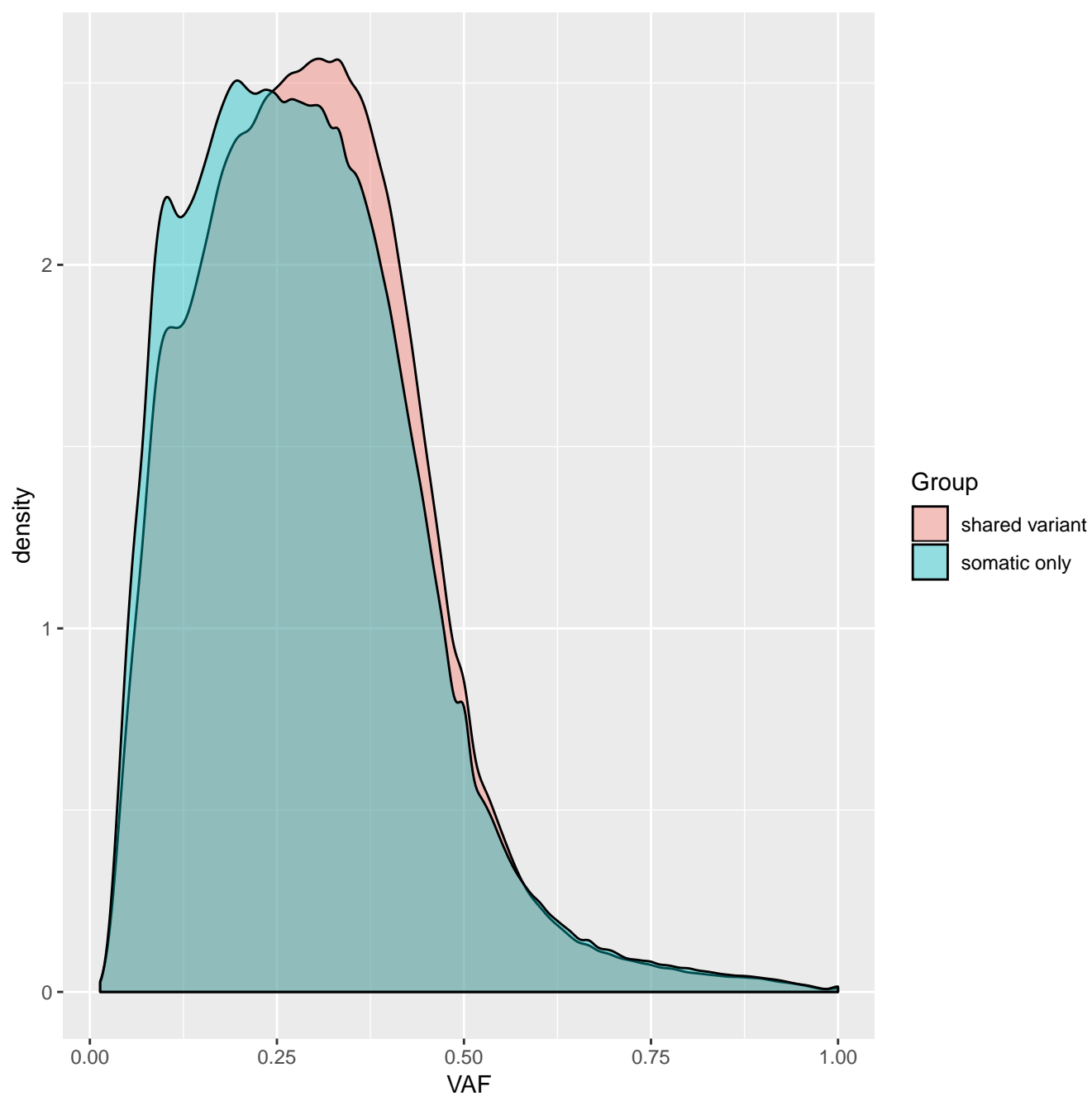

Supplement: Supplementary file 2 — Additional file 2: Supplemental Figure 2. The somatic variant allele frequency (VAF) distribution of somatically-unique and germline-shared somatic variants. [file 12859_2020_3508_MOESM2_ESM.pdf]

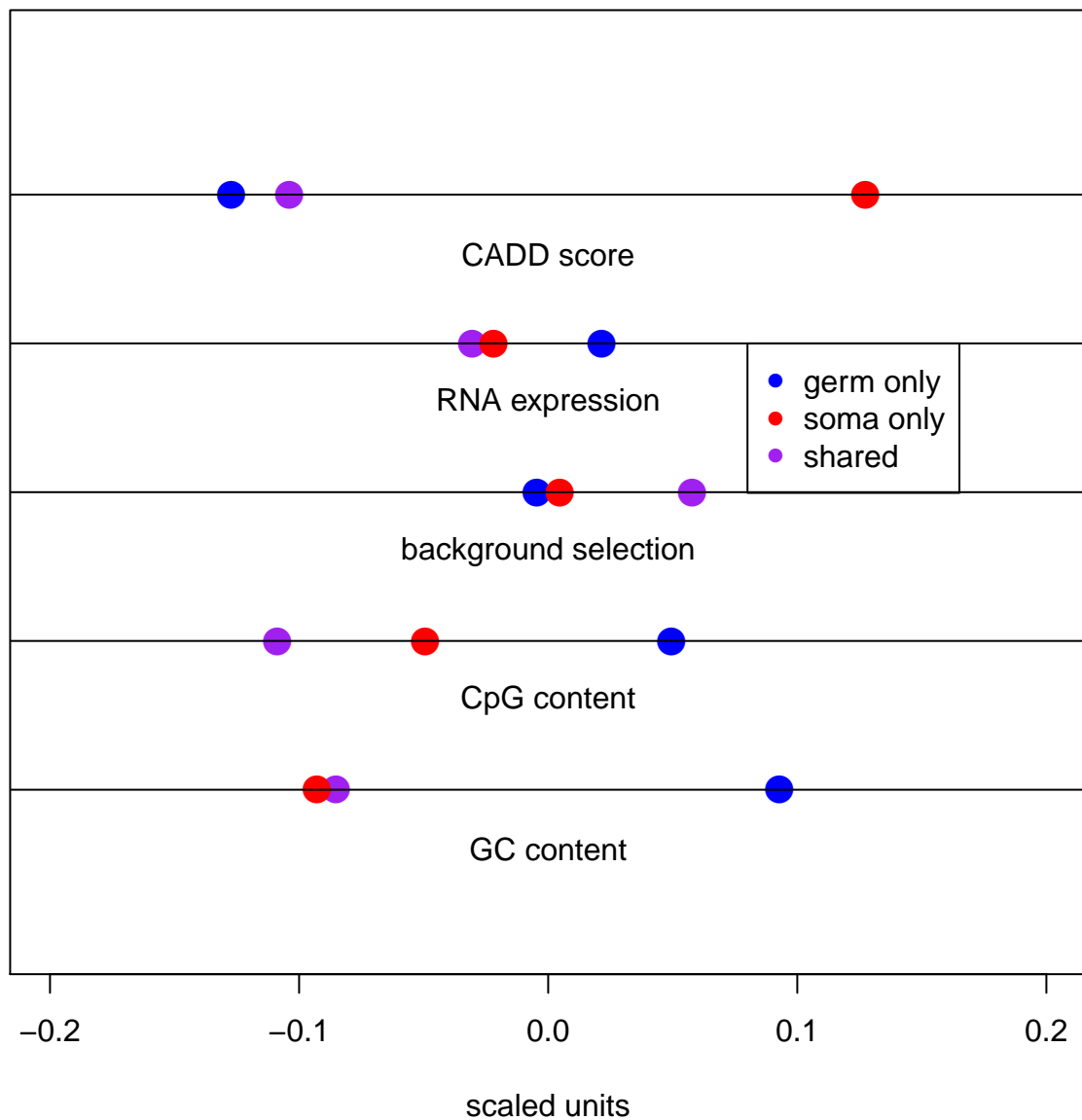

Supplement: Supplementary file 3 — Additional file 3: Supplemental Figure 3. Comparison of shared variants with somatically-unique and germline-unique variants along a range of genomic dimensions. Depending on the genomic dimension studied, shared variants better resemble either somatic values or germline values – or behave their own way. Units for each variable are scaled to make the set of all variants that are either somatic-unique or germline-unique have mean 0 and standard deviation 1. [file 12859_2020_3508_MOESM3_ESM.pdf]

Shared variant rate w Illumina germline

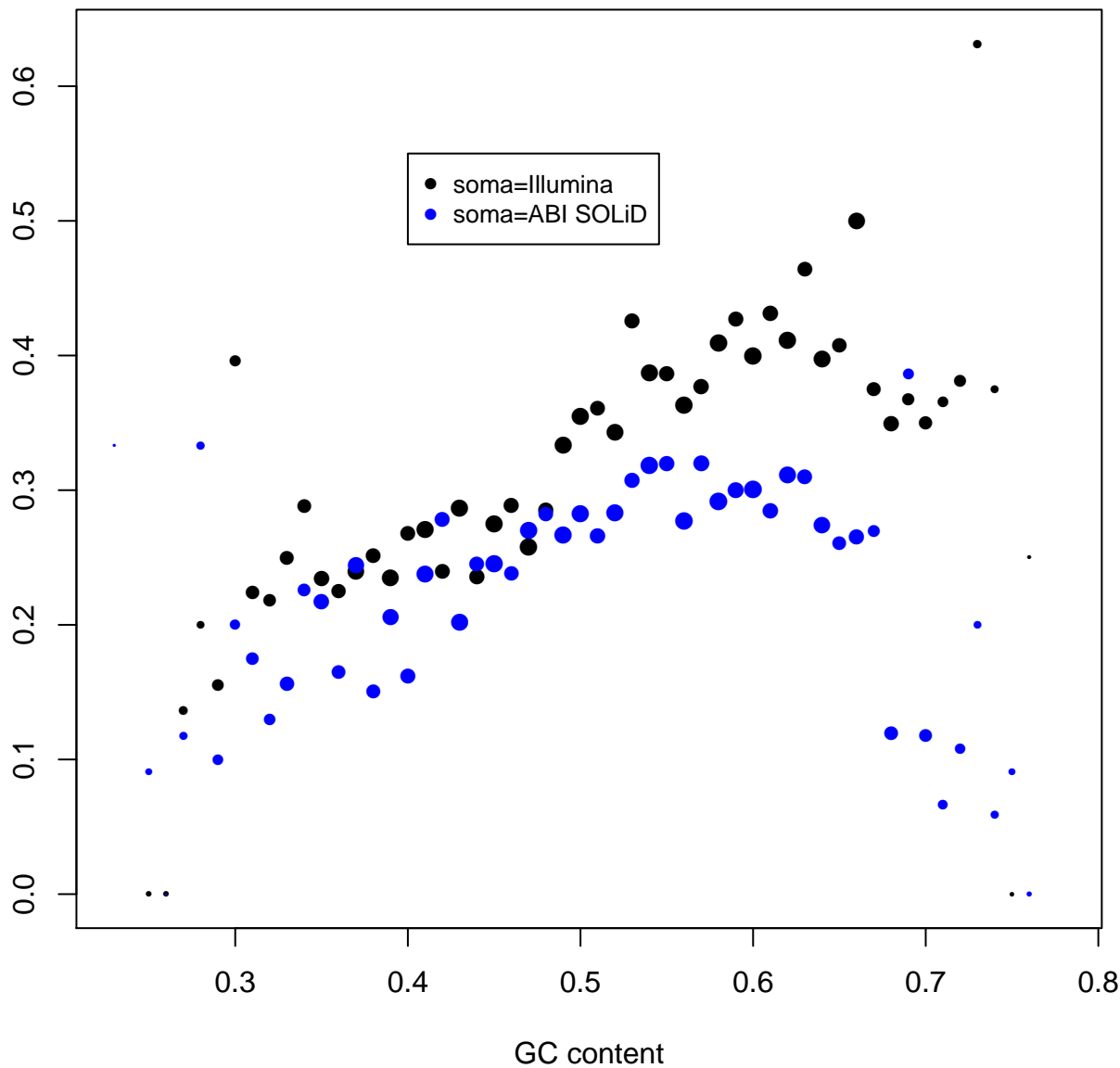

Supplement: Supplementary file 4 — Additional file 4: Supplemental Figure 4. Somatic sequencing platform and germline-shared variant rates. The rates at which somatic variants from 52 ABI-SOLiD sequenced colorectal tumors and 380 Illumina sequenced colorectal tumors are shared with an Illumina sequenced germline database, stratified by GC content bin. The size of each point is proportional to the logarithm of the number of variants that fall within each GC content bin. [file 12859_2020_3508_MOESM4_ESM.pdf]
